# Associations between attentional disengagement from distressed infant faces and cortisol reactivity are moderated by depressive symptoms in pregnant women: an eye-tracking study

**DOI:** 10.1007/s00737-025-01638-2

**Published:** 2026-01-03

**Authors:** Christine Dworschak, Gabriela Paganini, Abigail Beech, Kelley E. Gunther, Helena J. V. Rutherford, Jutta Joormann, Reuma Gadassi-Polack

**Affiliations:** 1 Department of Psychology, University of Zurich, Zurich, 8050 Switzerland; 2 Department of Psychology, Yale University, New Haven, CT 06520 USA; 3 Child Center for Anxiety and Related Disorders, Boston University, Boston, MA 02215 USA; 4 Neuroscience and Cognitive Science Program, University of Maryland College Park, Maryland, MD 20742 USA; 5 Faculty of Education and Gonda Multidisciplinary Brain Research Center, Bar-Ilan University, Ramat Gan, 5290002 Israel; 6 Child Study Center, Yale University, New Haven, CT 06520 USA; 7 Psychiatry Division, Tel Aviv Sourasky Medical Center, Tel Aviv, 6423906 Israel

**Keywords:** Depression, Attention, Infant distress, Cortisol, Pregnancy

## Abstract

**Purpose:**

Antenatal depression is a common psychological condition in pregnancy that negatively influences parenting. Theoretical models suggest that infant cue processing may represent one pathway by which depression may influence parenting. However, current understanding of how infant cue processing is linked with parenting in depression remains limited. Drawing upon previous research, cortisol stress reactivity may play an important role in this regard. The aim of the present study was to investigate the interaction between depressive symptoms and attentional disengagement from infant cues on cortisol reactivity. We examined this in a sample of pregnant and nulliparous women to test whether potential effects were specific to pregnancy.

**Methods:**

*N* = 79 women (*n* = 36 pregnant) completed two eye-tracking tasks examining disengagement from adult and infant stimuli, a stress manipulation task including collection of salivary cortisol, and filled out the BDI-II.

**Results:**

Pregnant (vs. nulliparous) women showed a stronger cortisol reactivity in response to a stress test. Additionally, a significant association between disengagement from distressed infant faces and cortisol reactivity was found in the pregnant (but not nulliparous) group, which was moderated by depressive symptoms. For pregnant women with low levels of depressive symptoms, a slower disengagement from distressed infant stimuli predicted a weaker cortisol reactivity in response to stress, while the opposite pattern was observed for pregnant women with high levels of depressive symptoms.

**Conclusions:**

Findings of the present study point at maternal distress during processing of infant distress as a potential intervention target for mothers with depression.

**Supplementary Information:**

The online version contains supplementary material available at 10.1007/s00737-025-01638-2.

## Highlights

• Pregnant (vs. nulliparous) women showed a stronger cortisol stress reactivity.

• Depression moderated the link between disengagement from infant distress and cortisol reactivity in pregnant women.

• For pregnant women high on depression, slower disengagement predicted stronger cortisol reactivity.

• For pregnant women low on depression, slower disengagement predicted weaker cortisol reactivity.

• Maternal distress in response to infant distress could offer an intervention target for mothers with depression.

**Supplementary Information:**

The online version contains supplementary material available at 10.1007/s00737-025-01638-2.

## Introduction

Pregnancy is a period characterized by profound physiological and psychological changes (Cárdenas et al. 2020). Although most women adapt to these changes, pregnancy is also considered a time of increased vulnerability for the onset or recurrence of psychiatric disorders, including depression (Smith et al. 2011). Indeed, antenatal depression is one of the most common disorders with pooled prevalence rates of 20.7% (Yin et al. 2021). The negative consequences of antenatal depression are far-reaching, affecting both mother and infant (e.g., Rutherford and Joormann 2017; Stein et al. 2014).

Previous research has been devoted to better understanding the link between maternal depression and poor child outcomes. In this context, parenting behavior has been described as a key pathway, by which maternal depression exerts its negative effects on child development (DeJong et al. 2016). For example, parents with depression have been found to show an increased tendency to withdraw or to engage in intrusive parenting behaviors (Murray et al. 2010). Similarly, research has shown that mothers with high levels of depressive symptoms seem to engage less in touching behavior (Mantis et al. 2019). Additionally, maternal depression has been linked to reduced maternal synchrony (Granat et al. 2017). However, despite evidence for the negative impact of parental depression on parenting, less is known about the mechanisms through which this association unfolds. As indicators of parenting already develop during pregnancy (Maas et al. 2016), this period offers a critical window for examining mothering and its determinants early on.

### The information processing model of perinatal depression and parenting

According to the information processing model by DeJong an colleagues (2016), one of the key mechanisms by which depression has been theorized to affect parenting behavior is infant cue processing. More specifically, the model suggests that cognitive processes altered in depression, such as rumination, may impair the processing of infant cues, affecting attention to such stimuli. Ultimately, difficulties in accurately perceiving infant cues are proposed to hinder parent-infant interactions, specifically the capacity and ability of contingent responsiveness. While the model provides an important starting point and a helpful overarching framework, a more fine-grained understanding of the processes specifically linking infant processing with parenting in the context of depression remains limited.

In the present work, we address this gap and aim to gain a more in-depth understanding of the role of infant cue processing in parenting in the context of depression. More specifically, we propose that infant cue processing – particularly of distress cues – is associated with increased cortisol stress reactivity for expecting mothers with depression, thereby pointing to a potential pathway through which maternal depression may contribute to more avoidant and less sensitive responding. In the following, we will first review the literature on maternal attention and depression, integrating insights from studies on both infant and adult cues, before introducing cortisol reactivity as a key variable and ultimately discussing the potential interplay between these variables in the context of depression.

### Maternal attention

As highlighted by the model (DeJong et al. 2016), maternal attention guides and shapes parenting behavior. More specifically, the literature conceptualizes maternal attention as a key process contributing to the development of maternal sensitivity (e.g., Pearson et al. 2011), which can be defined as the ability to respond to an infant’s cues in an adaptive and flexible manner (Ainsworth et al. 1978) and begins to develop during pregnancy (Maas et al. 2016; Rutherford and Joormann 2017). Maternal sensitivity is typically understood as a dynamic process characterized by three components, namely perception, interpretation, and active response to the infant’s cues (Ferrey et al. 2016; Shin et al. 2008). In this context, maternal attention would be considered a perceptual component of maternal sensitivity, thereby representing one of the initial stages in the behavioral responding process to the infant.

Pregnancy has been found to be associated with an attentional bias in the processing of infant (vs. adult) stimuli – here defined as a slower disengagement of attention from visual cues. Indeed, research shows that whereas in the context of non-pregnant individuals and adult stimuli an attention bias towards sad cues has been found to be rather maladaptive (e.g., Sanchez et al. 2013), preliminary evidence indicates the opposite pattern for pregnant women in the unique case of distressed infant stimuli. Indeed, studies have found that this attention bias towards distressed infant faces in pregnant women predicts better mother-child relationships (Pearson et al. 2011), suggesting more of an adaptive function of this bias during pregnancy.

Depression, however, seems to disrupt this bias in pregnant women. Indeed, in a study by Pearson and colleagues (2010), depressed pregnant women were found to show no attention bias towards distressed infant cues. Further, in a study by Xu and colleagues (2023), no prolonged gaze duration to the distressed infant cue was observed in the depressed (vs. non-depressed) pregnant group. Similarly, in a study by Rutherford and colleagues (2016), pregnant women with elevated levels of depression symptoms were found to have an attenuated P300 (a neural event-related potential (ERP) component linked to attention allocation) in response to distressed infant faces. Taken together, these findings suggest that depression seems to disrupt the observed adaptive attention bias towards distressed infant stimuli associated with pregnancy. However, current understanding of the processes linking maternal attention with parenting in the context of depression is still limited.

### Cortisol reactivity during pregnancy

Central parenting theories (e.g., Eisenberg’s Emotion Socialization Model, Eisenberg et al. 1998; Parental Meta-Emotion Philosophy, Gottman et al. 1996) highlight the crucial role of parents’ experienced distress in response to their child’s distress in parenting. Supporting these theories are empirical studies pointing at mothers’ physiological stress response to their infant distress as a significant predictor of maternal responding. For example, Leerkes and colleagues (2015) found mothers physiological arousal and regulation in response to their child crying to be predictive of their ability to respond to their infant in a sensitive manner. Indeed, higher arousal accompanied by lower regulation in response to child distress was linked to reduced maternal sensitivity. Similarly, a study by Zhang and colleagues (2021) showed that higher parental sympathetic arousal was associated with less supportive responding to children’s negative affect. Further, Leerkes et al. (2023) found high physiological arousal to be related to higher detachment when the level of regulation was low. Importantly, in all these studies physiological arousal was measured using skin conductance levels. However, given the crucial role of cortisol in depression (e.g., Nandam et al. 2020) and stress responding (e.g., James et al. 2023), investigating cortisol reactivity as a measure of physiological arousal may provide more fine-grained insights into the links between depression and parenting.

The hypothalamus-pituitary-adrenal (HPA) axis and its hormone cortisol are crucial for modulating the body’s stress response. Cortisol stress reactivity refers to the change in cortisol levels in response to a stressor, typically characterized by a rapid increase followed by a subsequent decline (Zorn et al. 2017). Pregnancy has been found to be linked to several changes in cortisol functioning including elevated basal cortisol levels as well as changes in the slope of the circadian rhythm of cortisol over the course of pregnancy (Almanza-Sepulveda et al. 2020). Additionally, changes in cortisol reactivity have been found to be related to the development of depression during pregnancy and postpartum, with stronger stress responses being linked to a higher risk for depression (Seth et al. 2016).

A main limitation of previous work investigating pregnancy-related changes in cortisol reactivity is the lack of a non-pregnant control group. From a methodological standpoint, including such a comparison is essential to drawing any valid conclusions about changes specific to pregnancy. Otherwise, observed differences may reflect individual variability or other confounding factors unrelated to pregnancy, rather than “true” effects of pregnancy (Beech et al. 2023; Fiterman and Raz 2019).

So far, only four studies have investigated differences in salivary cortisol reactivity in response to a stressor comparing pregnant and non-pregnant women. Conducting a hand cold pressor test, Kammerer and colleagues (2002) observed a significant cortisol stress response in non-pregnant, but not pregnant individuals. A study by Nierop and colleagues (2006) did not find any significant differences in cortisol reactivity between groups in response to the Trier Social Stress Test (TSST). Similarly, in a study by De Weerth and colleagues (2007) administering a public speaking and mental arithmetic test to pregnant and a modified test version to non-pregnant participants, a similar cortisol reactivity was found between groups. Moreover, Entringer and colleagues (2010) did not find a significant cortisol stress response in either pregnant or non-pregnant individuals in response to the TSST. Thus, results seem to be inconclusive and either point at no differences or a diminished stress response in pregnancy.

### The interplay between maternal attention, cortisol reactivity, and depression

Research has shown that cortisol reactivity is associated with both depression and maternal responding. Indeed, there is evidence pointing at individuals with depressive symptoms showing a stronger cortisol reactivity in response to stress (e.g., Murphy et al. 2015; Nandam et al. 2020; Zorn et al. 2017). Regarding associations with maternal responding, no study has yet investigated links between cortisol reactivity and maternal attention specifically. However, prior work on associations between general maternal parenting behavior – a construct distinct from, yet related to attention (Shin et al. 2008) - and cortisol reactivity can inform the current investigation. Here, studies have shown that a reduced degree of maternal sensitivity – as indicated by the degree of behavioral synchrony in a mother-infant-interaction paradigm - seems to be related to higher cortisol reactivity (Thompson and Trevathan 2008). Similarly, a study by Kiel and Buss (2013) found that more intrusive parenting behavior was linked to higher maternal cortisol reactivity.

Thus, maternal attentional processing of infant cues may be linked to cortisol reactivity with depressive symptoms moderating this association. More specifically, attentional processing of distressed infant cues may relate to stress responses in pregnant women differently depending on the level of depressive symptoms. That is, in pregnant women with high levels of depressive symptoms, a slower disengagement may be linked to a stronger cortisol reactivity, while the opposite pattern may be observed in those with low levels; thereby pointing to a potential pathway of how infant cue processing might affect parenting in the context of depression. This is supported by previously discussed research (a) indicating that an attention bias toward distressed infant cues is generally adaptive in the context of pregnancy (i.e., linked to healthy functioning and better mother-child-relationships; Pearson et al. 2010, 2011), but disrupted in antenatal depression, and (b) linking cortisol reactivity with both depression and maternal responding. However, to the best of our knowledge, no study has yet investigated this.

### The present study

We aimed to investigate (a) cortisol reactivity in pregnancy as well as (b) associations between attentional processing of infant cues (i.e., disengagement from distressed infant cues), cortisol reactivity, and antenatal depressive symptoms in a sample of pregnant and non-pregnant (i.e., nulliparous) women. To assess the specificity of a potential pattern of effects to infant (vs. adult) cues, we included a version of the eye-tracking task with adult stimuli. Specifically, we tested the following hypotheses (e = explorative; *=preregistered; preregistration: 10.17605/OSF.IO/HCXBG): First, we hypothesized that pregnant women will have a diminished cortisol reactivity in response to the TSST compared to nulliparous women. Second, we expected associations between attentional disengagement from sad adult stimuli, depression, and cortisol reactivity (e*). This was based on preliminary work by Sanchez and colleagues (2013) pointing at associations between slower disengagement from sad adult stimuli and stronger cortisol reactivity in depressed (vs. non-depressed) individuals only; however, as research in this field is limited, we kept this hypothesis exploratively, without a clear directional prediction. Third, for pregnant women only, we hypothesized that cortisol reactivity will be predicted by disengagement from distressed infant stimuli, such that slower disengagement from infant distressed faces will predict a weaker cortisol reactivity (*). Fourth, we expected that this association will be moderated by depression (e*) with a slower disengagement being related to a stronger (vs. weaker) cortisol reactivity in pregnant women with high (vs. low) levels of depressive symptoms.

## Materials and methods

This study is part of a larger project (e.g., Beech et al. 2023); only relevant procedures and measures are described.

### Participants

Pregnant and nulliparous participants were recruited using flyers, online advertisements and referrals from other labs. Individuals were included in the study if they (a) were at least 18 years old, (b) were fluent in English, (c) had normal or correct-to-normal vision, and (d) were of child-bearing age (maximum age of 45 years). Pregnant participants had to be in their second or third trimester of pregnancy and nulliparous women were included if they had no biological or adopted children and were older than 23 years. Age limitation of the nulliparous group was chosen to reduce demographic differences between the study groups. Participants received $30 for their participation.

A sample of 92 participants completed both the eye-tracking and stress task. A total of *n* = 13 participants had to be excluded resulting in an analytical sample of *n* = 79 participants (*n* = 36 pregnant and *n* = 43 nulliparous). See the supplementary material (Appendix A) for details on all exclusions made.

Regarding pregnancy status, 61.11% of the pregnant participants were primiparous with a mean gestational age of 28.78 weeks (SD = 6.32). Table 1 shows the demographic characteristics of the sample.

**Table 1:** Demographic characteristics for pregnant and nulliparous women

Demographic characteristic		Pregnant participants (*n* = 36) *n* (%) M (SD)	Nulliparous participants (*n* = 43) *n* (%) M (SD)	Test statistics
Age		30.86 (4.92)	28.09 (5.42)	*t*(76) = 2.38*
Race				χ^2^(3) = 1.70
	White/Caucasian	25 (69.44%)	32 (74.42%)	
	African American	5 (13.89%)	4 (9.30%)	
	Asian	5 (13.89%)	7 (16.28%)	
	Native American	0		
	Middle Eastern/Arab	1 (2.78%)		
Ethnicity				χ^2^(1) = 0.41
	Hispanic/Latina	5 (13.89%)	3 (6.98%)	
	Not Hispanic/Latina	31 (86.11%)	40 (93.02%)	
Education				χ^2^(3) = 0.28
	Less than high school	2 (5.56%)	3 (6.98%)	
	High school	0	0	
	Some college	4 (11.11%)	5 (11.63%)	
	Associate’s degree	0	0	
	Bachelor’s degree	29 (80.56%)	33 (76.74%)	
	Professional degree	1 (2.78%)	2 (4.65%)	
Marital Status				χ^2^(2) = 31.83***
	Single	8 (22.22%)	35 (81.40%)	
	Married	27 (75.00%)	6 (13.95%)	
	Divorced	0	2 (4.65%)	
Pregnancy	Pregnancy week	28.78 (6.32)		
	Primiparous	22 (61.11%)		

Note. *p <.05; ***p <.001

### Procedure

Participants were invited to a single lab session. The full procedure of the session is depicted in Figure S1 in the supplement.

### Tasks

#### Eye-tracking task

To assess attentional disengagement, participants completed an eye-tracking task developed by Sanchez and colleagues (2013), that in its original form focused on adult stimuli only. We used two variations of this task: an adult and an infant version. For each version of the task, participants completed three different types of trials: natural viewing trials, attentional engagement trials, and attentional disengagement trials. Given we were only interested in attentional disengagement, the following descriptions focus on this type of trial as well as the most important aspects only. A detailed description of the entire task can be found in the supplementary material (Appendix C).

### Adult version

#### Stimuli

Each stimulus was composed of two pictures with one showing a face with a neutral expression and another showing a face with an emotional expression of the same individual. Face images were selected from the Karolinska Directed Emotional Faces (KDEF) database (Lundqvist et al. 1998) and slightly edited. A total of 108 pairs of face images were used as stimuli consisting of 36 sad, 36 happy and 36 angry faces.

#### Disengagement trials

Figure S2 in the supplementary material depicts the sequence of the task. Within each trial, participants were shown a white screen for 500 ms, followed by a fixation cross for an additional 500 ms. After the presentation of the fixation cross, a random number was presented at the center of the screen for 1,000 ms and participants were instructed to say the number aloud as quickly as possible in order to ensure their eye gaze was in the middle of the screen prior to stimulus presentation. After that, a pair of two faces (happy-neutral, sad-neutral, angry-neutral) were presented for 3,000 ms side by side on the screen and the eye tracker waited for a fixation of at least 100 ms on the emotionally-valenced face. The circle or square then surrounded the neutral face and participants were instructed to respond with a key press whether the face was surrounded by a square or circle as quickly as possible. The required response was used in these trials to effectively disengage participants’ attention away from the emotionally-valenced face to the neutral face.

#### Eye-tracking apparatus 

Participants’ eye movements were recorded using a Tobii TX-120 eye tracking system, and eye gaze was calibrated before the start of the task.

#### Attention indices

Recorded eye movements were converted to visual fixation data using Tobii Studio software. In this study, we were interested in attentional disengagement defined as the time it took for participants to shift their gaze from the emotional face to the neutral face after it was surrounded by either a square or circle (stage 6 in Figure S2). Included in these times was the amount of time that participants spent attending to the stimuli during the ‘wait for fixation’ period, in addition to the time it took participants to shift their gaze from the initially fixated face to the face surrounded by the square or circle. For our analyses, we used average scores for attentional disengagement for each emotion condition for each participant.

### Infant version

To assess attentional disengagement from infant faces, we used the same procedure as for the adult task developed by Sanchez and colleagues (Sanchez et al. 2013) but adapted it by using infant facial stimuli and only two (instead of three) different emotional conditions (happy and distressed faces).

#### Stimuli

Infant face stimuli were extracted from a database consisting of digital photographs of 27 infants (Kringelbach et al. 2008) and were slightly edited. 66 pairs of face images were used consisting of 33 happy and 33 distressed faces (which were always presented next to a face with a neutral expression of the same individual).

#### Stress manipulation task

Individuals underwent a modified version of the Trier Social Stress Test (TSST; Kirschbaum et al. 1993). The TSST typically includes two tasks, the preparation and delivery of a speech as well as a mental arithmetic task. In the present study, the TSST was modified in two ways, namely that (1) participants were only asked to prepare but not actually give the speech as well as (2) that the topic of the speech was adapted by asking participants to give a talk on why they would be a good mother (see also Beech et al. (2023) for more details).

During the laboratory session, individuals were asked to provide saliva samples at four different time points, namely (1) after watching a relaxing video (approx.10 min), (2) after completion of the eye-tracking task (approx. 30 min), (3) immediately after the TSST (approx. 15 min), (4) 20 min later after watching a relaxing video and collecting all material (see Appendix D of the supplement for Figure S3 as well as for more details; the procedure has also been described in Beech et al. 2023). Although it is recommended to conduct cortisol reactivity measurements in the afternoon, we conducted sessions at all hours of the day to decrease participant burden and facilitate recruitment. Therefore, in both groups lab sessions were conducted at different times of day with start times varying between morning, midday and afternoon. There was no significant difference between groups in time of day (χ^2^(2) = 1.65, *p* =.44). Given the variability in time of day across sessions and its impact on baseline cortisol levels (Pruessner et al. 1997), we decided to use the area under the curve with respect to increase (AUCi; Pruessner et al. 2003) as opposed to ‘0’ (AUCg) as a measure for cortisol reactivity. See Appendix D of the supplement for the formula on which the calculation of AUCi scores was based.

As a manipulation check, individuals were asked to rate their negative affect at each saliva sample collection time point using five emotions (*upset*,* tense*,* sad*,* irritable*,* nervous*) rated on a scale from 0 to 10 with higher scores indicating stronger experience of this emotion. Cronbach’s alpha ranged between 0.69 and 0.77 for the four assessment time points.

### Questionnaires

#### Depressive symptoms

To assess depressive symptoms, we used the 21-item Beck Depression Inventory-II (BDI-II, Beck et al. 1996). Cronbach’s alpha was 0.86. In our sample, sum scores ranged between 0 and 25 (out of a possible range of 0–63) with an overall mean sum score of M = 7.00 (SD = 5.68). Based on interpretative guidelines by Beck and colleagues (1996), out of a total of *n* = 69 participants responding to the BDI-II, 60 (87%) participants reported minimal or no depression, 6 (9%) mild depression, and 3 (4%) moderate depression. Nine participants (13%) met the criterion for depression (minimum sum score of 14 according to Beck et al. (1996).

#### Cortisol confounders

To account for the impact of potential confounding variables on cortisol assessments, participants reported on potential confounders (e.g., Tobacco use) at the beginning of the session. Additionally, participants were asked to refrain from eating or drinking anything during the session.

#### Additional questionnaires

 measures of psychopathology to better assess the psychiatric status of participants. A list of these measures as well as respective descriptives can be found in Appendix E in the supplementary material.

### Analytical procedure

Data were analyzed using the statistic software R Version 4.2.2 (R Core Team 2022). First, we checked whether our stress manipulation was successful by analyzing differences between negative affect ratings and cortisol levels collected after vs. before the stress induction using paired-sample *t*-tests. Here, person-mean-centered scores were used for cortisol levels to account for baseline differences due to the different timing of lab sessions. After that, we tested for group differences between pregnant and nulliparous women on all relevant study variables and investigated the potential influence of cortisol confounders on AUCi cortisol levels using independent-sample *t*-tests (no significant differences, see supplement Appendix D). Further, given that outlier removal has been described as an important step in the cleaning process of eye-tracking data (e.g., Eskenazi 2023; Godwin et al. 2021), we excluded extreme outliers (above/below two and a half standard deviations from the mean) for the respective variable of each emotion condition of each version of the eye-tracking task. This resulted in the exclusion of one to three participants for the different emotion conditions of the adult task and one to five individuals for the infant task.

For the first hypothesis, we used an independent-samples *t*-test to investigate differences in cortisol reactivity between pregnant and nulliparous women. To test our second hypothesis, linear regression models were estimated. Analyses were conducted using the full sample (i.e., including pregnant and nulliparous women). In regression models, attentional disengagement from sad adult stimuli, depressive symptoms (BDI-II sum scores) as well as their interaction were added as independent variables; cortisol reactivity (AUCi) was entered as the dependent variable. Simple slope analyses were used to investigate significant interaction effects. Both BDI-II and attentional disengagement scores were mean-centered to make interpretation of intercepts clearer as well as to mitigate multicollinearity. The same models were run with disengagement from angry and happy adult faces. To test our third and fourth hypotheses, we followed the same procedure as for the second hypothesis. The only differences from the models used for the second hypothesis were that depressive symptoms (as well as their interaction with attentional disengagement) were only entered into the model in a second step as well as that analyses were ran separately for each group. We conducted sensitivity analyses for the models used for hypotheses 2–4 controlling for time of day of lab sessions; results of interest did not change and can be found in Appendix F in the supplement. Data and code are available on OSF (https://osf.io/uedy6/?view_only=09a716d9f70d4423a302ae41deb36fdb).

## Results

### Manipulation check TSST

Negative affect ratings at time point 3 (right after the TSST) were significantly higher than at baseline measurements time point 1 (*M*_*time_1*_=1.00 (*SD*_*time_1*_=1.14); *M*_*time_3*_=1.75 (*SD*_*time_3*_=1.48); *t*(74)=−5.00, *p* <.001) and time point 2 (*M*_*time_2*_=0.95 (*SD*_*time_2*_=0.97); *t*(73)=−6.47, *p* <.001). Additionally, there was a significant increase in cortisol levels from baseline assessment time point 2 to time point 4 (20 min after the TSST; *M*_*time_2*_= −0.36 (*SD*_*time_2*_=1.02); *M*_*time_4*_=0.14 (*SD*_*time_4*_=1.63); *t*(78)=−2.29, *p* <.05). Thus, the stress manipulation was successful.

### Group differences in study variables

Table 2 displays means and standard deviations of all study variables by group along with *t*-tests examining differences between the groups. We did not find any significant differences between groups except for cortisol reactivity (see below). See also Appendix G in the supplement for zero-order correlations.

**Table 2:** Study variables for pregnant and nulliparous participants

Variable		Pregnant participants (*n* = 36) M (SD)	Nulliparous participants (*n* = 43) M (SD)	Test statistics
Cortisol Reactivity to TSST	AUCi cortisol	9.53 (127.53)	−68.80 (149.51)	*t*(77) = 2.51, *p* =.014*, *d* = 0.56
Disengagement from Adult faces	Sad	0.34 (0.11)	0.34 (0.11)	*t*(62)=−0.13, *p* =.895, *d* = 0.03
	Angry	0.32 (0.11)	0.35 (0.24)	*t*(60)=−0.70, *p* =.489, *d* = 0.15
	Happy	0.31 (0.14)	0.33 (0.14)	*t*(68)=−0.42, *p* =.677, *d* = 0.10
Disengagement from Infant faces	Distressed	0.35 (0.24)	0.37 (0.27)	*t*(71)=−0.31, *p* =.759, *d* = 0.07
	Happy	0.35 (0.24)	0.33 (0.13)	*t*(51) = 0.49, *p* =.625, *d* = 0.12
Depressive symptoms	BDI	8.42 (4.02)	5.84 (6.57)	*t*(63) = 2.00, *p* =.050, *d* = 0.46

Note. Attentional disengagement was measured in seconds; *p <.05

### Differences in cortisol reactivity between pregnant and nulliparous women

Unexpectedly, t-tests revealed a higher cortisol reactivity in pregnant compared to nulliparous individuals (*t*(77) = 2.51, *p* =.014, *d* = 0.56) (Table 2).

### Adult stimuli: moderation of the disengagement-cortisol reactivity link by depression

No significant associations between attentional disengagement from sad adult stimuli, depressive symptoms, and cortisol reactivity were found (Table 3). Models for all other emotion conditions (angry, happy) were also nonsignificant (Table 3).

**Table 3:** Results of linear regression models investigating associations between attentional disengagement from adult faces, depression, and cortisol reactivity in the full sample (N = 79)

	Predicted	Cortisol Reactivity			
		*B*	*SE*	*t*	95% *CI*
Sad faces	Intercept	−27.23	17.43	−1.56	−62.10, 7.64
	Disengagement	193.42	157.18	1.23	−121.09, 507.93
	Depressive symptoms	0.53	3.05	0.17	−5.58, 6.64
	Disengagement: Depressive symptoms	−23.00	31.36	−0.73	−85.75, 39.75
Angry faces	Intercept	−24.73	17.62	−1.40	−59.96, 10.50
	Disengagement	82.07	126.14	0.65	−170.17, 334.31
	Depressive symptoms	1.38	3.19	0.43	−5.00, 7.76
	Disengagement: Depressive symptoms	7.43	18.32	0.41	−29.21, 44.07
Happy faces	Intercept	−25.20	17.76	−1.42	−60.72, 10.33
	Disengagement	18.04	215.06	0.08	−412.15, 448.23
	Depressive symptoms	0.64	3.11	0.21	−5.57, 6.85
	Disengagement: Depressive symptoms	2.39	49.45	0.05	−96.54, 101.31

### Infant stimuli: moderation of the disengagement-cortisol reactivity link by depression

**Fig. 1:**
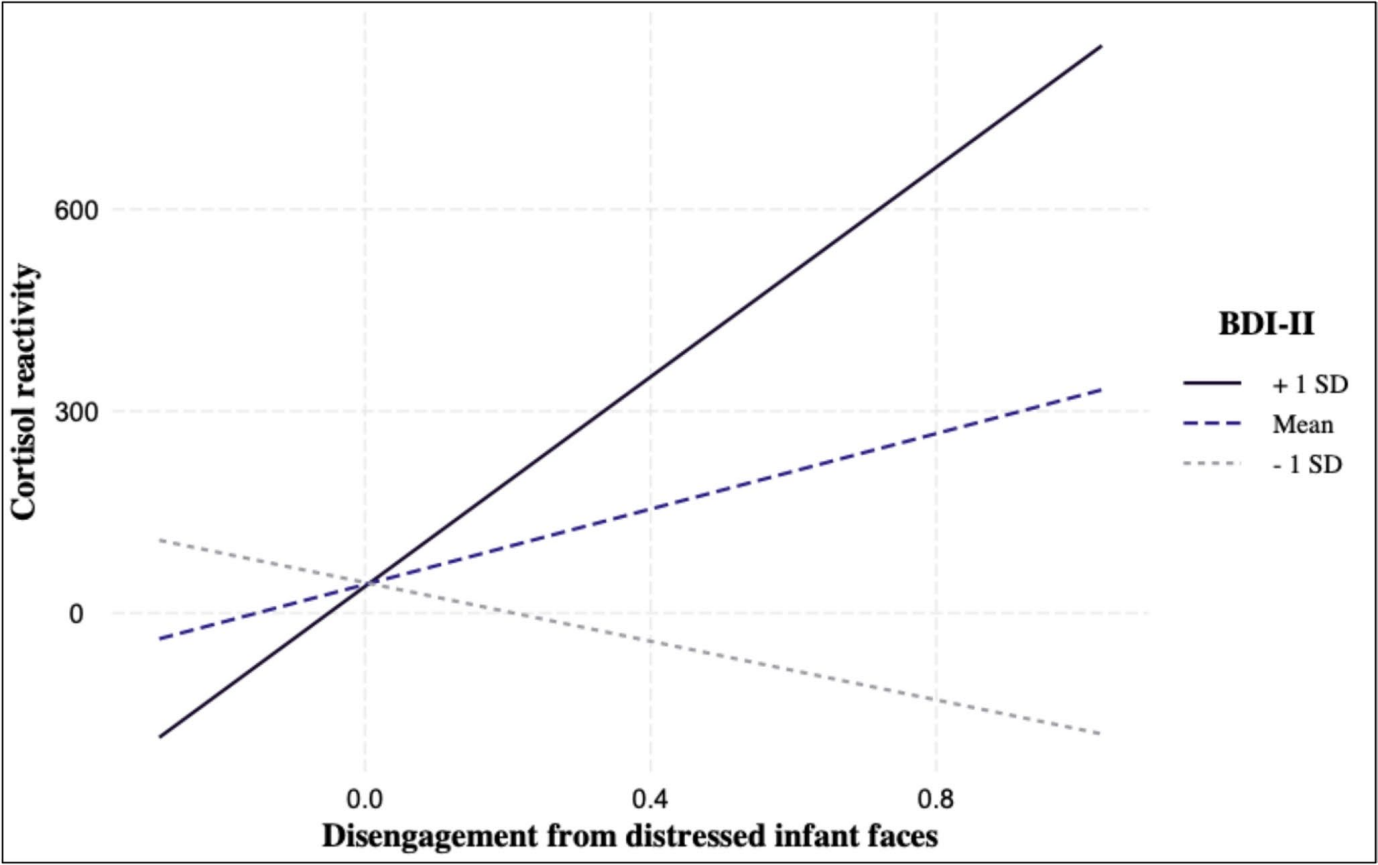
Interaction effect of disengagement from distressed infant faces and depressive symptoms (BDI-II) on cortisol reactivity in the pregnant group (*n* = 36)

As predicted, in the pregnant group only, a longer time to disengage from distressed infant faces was associated with lower cortisol reactivity in response to the TSST (Table 4). When depressive symptoms and their interaction with disengagement were added to the model, the interaction between depressive symptoms and disengagement from distressed infant faces was significant (Table 4). As can be seen in Fig. 1, simple slope analyses indicated that for pregnant individuals with low levels of depressive symptoms (1 SD below the Mean), a longer time to disengage from distressed infant faces was associated with a lower cortisol reactivity (*B*=−218.06, *SE* = 70.86, *p* =.005). The opposite pattern was observed for pregnant individuals with high levels of depressive symptoms (1 SD above the Mean), i.e., a longer time to disengage from distressed infant faces was associated with a higher cortisol reactivity (*B* = 778.70, *SE* = 349.40, *p* =.03). No significant effects were found in the models focused on happy infant faces. In the nulliparous group, none of the models revealed any significant effects (see Table 5).

**Table 4:** Results of linear regression models investigating associations between attentional disengagement from infant faces, depression, and cortisol reactivity in the pregnant group (n = 36)

	Predicted	Cortisol Reactivity				Cortisol Reactivity			
		Model: Step 1				Model: Step 2			
		*B*	*SE*	*t*	95% *CI*	*B*	*SE*	*t*	95% *CI*
Distressed faces	Intercept	4.72	20.42	0.23	−36.83, 46.26	43.47	21.60	2.01	−0.93, 87.87
	Disengagement	−199.92*	86.39	−2.31	−375.69, −24.15	113.42	133.14	0.85	−160.26, 387.10
	Depressive symptoms					−0.71	4.55	−0.16	−10.06, 8.65
	Disengagement: Depressive symptoms					122.12**	42.11	2.90	35.56, 208.69
Happy faces	Intercept	6.05	22.17	0.27	−39.10, 51.20	−1.40	24.25	−0.06	−51.34, 48.54
	Disengagement	125.35	92.44	1.36	−62.95, 313.64	81.80	119.15	0.69	−163.60, 327.19
	Depressive symptoms					2.15	5.54	0.39	−9.25, 13.55
	Disengagement: Depressive symptoms					−26.17	40.33	−0.65	−109.22, 56.89

Note. In Step 1 of the model, disengagement was entered as the only predictor. In Step 2, depressive symptoms and interaction of depressive symptoms and disengagement were added; *p <.05; **p <.01

**Table 5:** Results of linear regression models investigating associations between attentional disengagement from infant faces, depression, and cortisol reactivity in the nulliparous group (n = 43)

	Predicted	Cortisol Reactivity				Cortisol Reactivity			
		Model: Step 1				Model: Step 2			
		*B*	*SE*	*t*	95% *CI*	*B*	*SE*	*t*	95% *CI*
Distressed faces	Intercept	−63.53**	18.61	−3.41	−101.28, −25.78	−61.37**	21.57	−2.85	−105.49, −17.25
	Disengagement	47.43	68.80	0.69	−92.10, 186.97	60.62	87.68	0.69	−118.71, 239.95
	Depressive symptoms					−0.51	3.50	−0.15	−7.66, 6.64
	Disengagement: Depressive symptoms					11.08	29.10	0.38	−48.43, 70.59
Happy faces	Intercept	−63.08**	19.79	−3.19	−103.30, −22.85	−60.19	22.67	−2.66	−106.70, −13.68
	Disengagement	55.00	148.87	0.37	247.54, 357.53	75.57	168.48	0.45	−270.11, 421.26
	Depressive symptoms					−0.41	3.29	−0.13	−7.16, 6.34
	Disengagement: Depressive symptoms					29.44	29.41	1.00	−30.90, 89.78

Note. In Step 1 of the model, disengagement was entered as the only predictor. In Step 2, depressive symptoms and interaction of depressive symptoms and disengagement were added; **p <.01

## Discussion and conclusions

Aim of the present study was to examine (a) cortisol stress reactivity in pregnancy as well as (b) whether the link between attentional processing of infant cues and cortisol reactivity is moderated by antenatal depressive symptoms. We investigated this in a sample of pregnant and nulliparous women using both an eye-tracking paradigm as well as a social stress test.

Interestingly, we observed a stronger cortisol stress reactivity in response to the TSST in pregnant compared to nulliparous women. This finding is in the opposite direction of our hypothesis and prior studies, who either found no differences or a diminished cortisol response in pregnant compared to nulliparous women (De Weerth et al. 2007; Entringer et al. 2010; Kammerer et al. 2002; Nierop et al. 2006). However, these studies also have some shortcomings, including small sample sizes (Kammerer et al. 2002) as well as differing stress test procedures used between groups (De Weerth et al. 2007); limitations, that we tried to address in the current investigation. Additionally, it is important to consider that whereas in previous studies the topic of the speech task administered as part of the stress test was unrelated to the context of parenting, participants in our study were asked to give a talk on why they would be a good mother, which might also have contributed to the differing results. Indeed, studies demonstrate that differences between mothers and non-mothers are often only found when the stimulus is related to parenting (e.g., child vs. adult faces) (e.g., Thompson-Booth et al., 2014a, b). Thus, changes in stress response and related differences between pregnant and non-pregnant individuals may be uniquely tied to parenting-related topics; however, this remains to be further investigated, as we did not include a non-parenting speech condition in the current study. Taken together, findings of our study suggest that pregnancy appears to be associated with an increase in cortisol stress reactivity. Given that prior research has robustly documented the negative short- and long-term effects of prenatal maternal stress on maternal mental health (e.g., Beech et al. 2023) and on child development (for a review, see Graignic-Philippe 2014), a better understanding of the respective pregnancy-related changes in the HPA-axis is needed.

As predicted, in the current study we found that for pregnant women (but not for nulliparous women), a longer time to disengage from distressed infant stimuli was associated with a lower cortisol reactivity in response to the TSST. Importantly, this association was moderated by depressive symptoms, such that for pregnant individuals with high levels of depressive symptoms, a longer time to disengage from distressed infant faces was associated with a stronger cortisol stress reactivity, whereas the opposite pattern was observed for pregnant women with low levels of depressive symptoms.

Our findings suggest that mothers experiencing higher levels of depressive symptoms during pregnancy may show a high level of stress in response to a stressor, in our case the TSST. This stress response was linked to attention to infant stimuli, such that a higher level of stress was associated with a slower disengagement from distressed infant stimuli in pregnant women with higher depression symptoms. Possibly, this experience of heightened stress may be linked to a future tendency to avoid signals of infant distress, such as by withdrawing attention from one’s own child or attending to one’s child with less sensitivity. That is, avoidant strategies that a mother may use to regulate her own emotions may not be beneficial to her infant. Such an interpretation would need to be confirmed by further research examining these associations across the perinatal period. Nevertheless these findings add to central parenting theories (e.g., Eisenberg’s Emotion Socialization Model, Eisenberg et al. 1998; Parental Meta-Emotion Philosophy, Gottman et al. 1996) pointing at parental distress in response to child negative emotions as playing a key role for parenting as well as to findings demonstrating an increased tendency for withdrawing parenting behaviors in parents with depression (Murray et al. 2010).

To the best of our knowledge, this study is the first to investigate associations among attentional processing of infant cues, cortisol reactivity, and antenatal depression. One study that has examined associations between related constructs (physiological reactivity, maternal sensitivity) in a primiparous sample (Leerkes et al. 2015) found neither a direct effect of emotional risk (including depressive symptoms) nor an indirect effect via physiological arousal and regulation on maternal sensitivity. Our findings diverge from this prior investigation, as we found significant associations among attentional disengagement, cortisol reactivity, and depression. However, it is important to note that while in our study, we assessed attentional disengagement from distressed infant cues via eye-tracking as an indicator of maternal sensitivity, Leerkes and colleagues conducted an observational behavioral assessment of maternal sensitivity; a significant methodological difference, which may have contributed to the differing findings. More studies are needed to investigate whether associations between indicators of maternal sensitivity, stress reactivity, and depression are specific to the perceptual component of maternal sensitivity (i.e., attention) or whether they extend to actual responding behavior in mothers.

While in the present study we found an association between attentional processing of infant distress cues and cortisol stress reactivity, our findings do not shed light on the underlying mechanisms. Interestingly, other studies have also pointed at mothers’ beliefs and attributions of infant crying or distress as a key variable for maternal responding (Girod et al. 2023; Leerkes et al. 2022). For example, it has been found that the beliefs mothers have about infants crying (e.g., hostile reasons vs. emotional attributions) significantly influence maternal sensitive responding (Girod et al. 2023; Leerkes et al. 2022). Investigating mothers’ beliefs and attributions of infant crying as a potential mediator in the attention-stress relation may, therefore, present a promising avenue for future research.

Findings of the present study add to the existing literature on associations among maternal depression, parenting behavior, and negative child outcomes by highlighting the possible role of stress reactivity in the process. Integrated into the information processing model of DeJong and colleagues (2016), our results point at significant associations between infant cue processing and stress reactivity, thereby suggesting a potentially novel pathway through which parenting may be affected. Investigating the mediation of the attention-parenting link via stress reactivity could, thus, offer a valuable next step for future work in the field.

Interestingly, we found no effect of pregnancy status (pregnant vs. nulliparous) on attentional disengagement from distressed infant cues. Indeed, there was no significant difference in the time it took participants to disengage their attention from distressed to neutral faces between pregnant and nulliparous women. This finding diverges from previous studies demonstrating an increased attention towards distressed infant faces in pregnant women (e.g., Pearson et al. 2010, 2011; Thompson-Booth et al. 2014a). However, it is important to note that all these previous studies used reaction time measures instead of eye-tracking procedures to assess attentional processing. While reaction time measures are often criticized to only capture a brief snapshot of attention, eye-tracking methodologies allow for a more direct assessment of attentional allocation (Van Ens et al. 2019). Additionally, compared to the other studies, our sample was relatively small with a large variation in the pregnancy stage of participants, which may also have contributed to the differing findings. More research is needed to investigate whether the previously reported attention bias extends to more direct measurements of attentional allocation as well as to pregnancy in general rather than to specific stages.

### Clinical implications

The results of the present study have important implications to clinical practice. Our results point at maternal distress during processing of infant distress as a potential intervention target for mothers with depression. Mothers experiencing high levels of depressive symptoms could benefit from stress regulation interventions teaching them how to more effectively regulate their emotions (see also a related call to action in Penner and Rutherford 2022). One example of such an intervention is Minding the Baby, a program focused on promoting emotion regulation abilities by improving parental mentalization (Slade et al. 2020). Additionally, our study shows that associations between alterations in attentional processing and stress are already evident during pregnancy, suggesting that interventions should start early-on in order to buffer any negative effects on both mother and child.

### Limitations

The current study also has some limitations. First, our sample was small with a limited variance and frequency of depressive symptoms. Second, the heterogeneity among pregnant individuals regarding the stage of pregnancy and pregnancy duration may impair the validity of our results. Third, in the modified version of the TSST used in the current study participants were only asked to prepare but not actually give the speech, limiting our ability to significantly increase stress levels. Fourth, in our analyses, we did not specifically account for participants wo used steroids, which may have biased our findings. Fifth, our study design was cross-sectional, which does not allow to draw any conclusions about the direction of observed associations. Future experimental studies are needed to clarify the causality of these effects.

## Supplementary Information

Below is the link to the electronic supplementary material.

Supplementary material 1(DOCX 298 KB)

## Data Availability

Data and code are available on OSF (https://osf.io/uedy6/?view_only=09a716d9f70d4423a302ae41deb36fdb).

## References

[CR1] Ainsworth MDS, Blehar MC, Waters E, Wall S (1978) Patterns of attachment: A psychological study of the strange situation. Erlbaum

[CR2] Almanza-Sepulveda ML, Fleming AS, Jonas W (2020) Mothering revisited: a role for cortisol? Horm Behav 121:104679. 10.1016/j.yhbeh.2020.104679 31927022 10.1016/j.yhbeh.2020.104679

[CR3] Beck AT, Steer RA, Brown GK (1996) Manual for the beck depression inventory-II. Psychological Corporation

[CR4] Beech A, Edelman A, Yatziv T, Rutherford HJV, Joormann J, Gadassi-Polack R (2023) Cortisol reactivity to a laboratory stressor predicts increases in depressive symptoms in perinatal and nulliparous women during population-level stress. J Affect Disord 340:33–41. 10.1016/j.jad.2023.07.093 37499916 10.1016/j.jad.2023.07.093 PMC10529046

[CR5] Cárdenas EF, Kujawa A, Humphreys KL (2020) Neurobiological changes during the peripartum period: implications for health and behavior. Soc Cogn Affect Neurosci 15(10):1097–1110. 10.1093/scan/nsz091 31820795 10.1093/scan/nsz091 PMC7657461

[CR6] DeJong H, Fox E, Stein A (2016) Rumination and postnatal depression: a systematic review and a cognitive model. Behav Res Ther 82:38–49. 10.1016/j.brat.2016.05.003 27203622 10.1016/j.brat.2016.05.003 PMC4898208

[CR7] Eisenberg N, Cumberland A, Spinrad TL (1998) Parental socialization of emotion. Psychol Inq 9(4):241–273. 10.1207/s15327965pli0904_1 16865170 10.1207/s15327965pli0904_1 PMC1513625

[CR8] Entringer S, Buss C, Shirtcliff EA, Cammack AL, Yim IS, Chicz-DeMet A, Sandman CA, Wadhwa PD (2010) Attenuation of maternal psychophysiological stress responses and the maternal cortisol awakening response over the course of human pregnancy. Stress 13(3):258–268. 10.3109/10253890903349501 20067400 10.3109/10253890903349501 PMC2862645

[CR9] Eskenazi MA (2023) Best practices for cleaning eye movement data in reading research. Behav Res Methods 56(3):2083–2093. 10.3758/s13428-023-02137-x 37222925 10.3758/s13428-023-02137-x

[CR10] Ferrey AE, Santascoy N, McCrory EJ, Thompson-Booth C, Mayes LC, Rutherford HJV (2016) Motivated attention and reward in parenting. Parenting 16(4):284–301. 10.1080/15295192.2016.1184928

[CR11] Fiterman O, Raz S (2019) Cognitive, neural and endocrine functioning during late pregnancy: An Event-Related Potentials study. Horm Behav 116:104575. 10.1016/j.yhbeh.2019.104575 31442429 10.1016/j.yhbeh.2019.104575

[CR12] Girod SA, Leerkes EM, Zvara BJ (2023) Childhood maltreatment predicts maternal sensitivity to distress: negative attributions during the transition to parenthood. J Fam Psychol 37(5):709–719. 10.1037/fam0001088 37053420 10.1037/fam0001088 PMC10440301

[CR13] Godwin HJ, Hout MC, Alexdóttir KJ, Walenchok SC, Barnhart AS (2021) Avoiding potential pitfalls in visual search and eye-movement experiments: A tutorial review. Atten Percept Psychophys 83(7):2753–2783. 10.3758/s13414-021-02326-w 34089167 10.3758/s13414-021-02326-w PMC8460493

[CR14] Gottman JM, Katz LF, Hooven C (1996) Parental Meta-Emotion philosophy and the emotional life of families: theoretical models and preliminary data. J Fam Psychol 10(3):243–268

[CR15] Graignic-Philippe R (2014) Effects of prenatal stress on fetal and child development: A critical literature review. Neurosci Biobehav Rev 43:137–162. 10.1016/j.neubiorev.2014.03.022 24747487 10.1016/j.neubiorev.2014.03.022

[CR16] Granat A, Gadassi R, Gilboa-Schechtman E, Feldman R (2017) Maternal depression and anxiety, social synchrony, and infant regulation of negative and positive emotions. Emotion 17(1):11–27. 10.1037/emo0000204 27441576 10.1037/emo0000204

[CR17] James KA, Stromin JI, Steenkamp N, Combrinck MI (2023) Understanding the relationships between physiological and psychosocial stress, cortisol and cognition. Front Endocrinol 14:1085950. 10.3389/fendo.2023.1085950 10.3389/fendo.2023.1085950 PMC10025564 36950689

[CR18] Kammerer M, Adams D, Castelberg Bvon, Glover V (2002) Pregnant women become insensitive to cold stress. BMC Pregnancy Childbirth 2:8. 10.1186/1471-2393-2-8 12437774 10.1186/1471-2393-2-8 PMC137604

[CR19] Kiel EJ, Buss KA (2013) Toddler inhibited temperament, maternal cortisol reactivity and embarrassment, and intrusive parenting. J Fam Psychol 27(3):512–517. 10.1037/a0032892 23750532 10.1037/a0032892 PMC3817411

[CR20] Kirschbaum C, Pirke K-M, Hellhammer DH (1993) The ‘Trier social stress Test’ – a tool for investigating psychobiological stress responses in a laboratory setting. Neuropsychobiology 28(1–2):76–81. 10.1159/000119004 8255414 10.1159/000119004

[CR21] Kringelbach ML, Lehtonen A, Squire S, Harvey AG, Craske MG, Holliday IE, Green AL, Aziz TZ, Hansen PC, Cornelissen PL, Stein A (2008) A specific and rapid neural signature for parental instinct. PLoS One 3(2):e1664. 10.1371/journal.pone.0001664 18301742 10.1371/journal.pone.0001664 PMC2244707

[CR22] Leerkes E, Supple AJ, O’Brien M, Calkins SD, Haltigan JD, Wong MS, Fortuna K (2015) Antecedents of maternal sensitivity during distressing tasks: integrating attachment, social information processing, and psychobiological perspectives. Child Dev 86(1):94–111. 10.1111/cdev.12288 25209221 10.1111/cdev.12288 PMC5242093

[CR23] Leerkes E, Sommers S, Bailes L (2022) The validity of prenatal assessments of mothers’ emotional, cognitive, and physiological reactions to infant cry. Parenting 22(4):286–314. 10.1080/15295192.2021.1975122 36247411 10.1080/15295192.2021.1975122 PMC9565640

[CR24] Leerkes E, Girod SA, Buehler C, Shriver LH, Wideman L (2023) Interactive effects of maternal physiological arousal and regulation on maternal sensitivity: replication and extension in an independent sample. Dev Psychobiol 65(2):e22375. 10.1002/dev.22375 36811368 10.1002/dev.22375 PMC9972255

[CR25] Lundqvist D, Flykt A, Öhman A (1998) The karolinska directed emotional faces (KDEF). Department of Neurosciences Karolinska Hospital

[CR26] Maas AJBM, De Cock ESA, Vreeswijk CMJM, Vingerhoets AJJM, Van Bakel HJA (2016) A longitudinal study on the maternal–fetal relationship and postnatal maternal sensitivity. J Reprod Infant Psychol 34(2):110–121. 10.1080/02646838.2015.1112880

[CR27] Mantis I, Mercuri M, Stack DM, Field TM (2019) Depressed and non-depressed mothers’ touching during social interactions with their infants. Dev Cogn Neurosci 35:57–65. 10.1016/j.dcn.2018.01.005 29422337 10.1016/j.dcn.2018.01.005 PMC6968954

[CR28] Murphy SE, Braithwaite EC, Hubbard I, Williams KV, Tindall E, Holmes EA, Ramchandani PG (2015) Salivary cortisol response to infant distress in pregnant women with depressive symptoms. Arch Womens Ment Health 18(2):247–253. 10.1007/s00737-014-0473-0 25352317 10.1007/s00737-014-0473-0 PMC4516861

[CR29] Murray L, Halligan SL, Cooper P (2010) Effects of postnatal depression on mother-infant interactions and child development. In J. G. Bremner & T. D. Wachs (Hrsg.), *Handbook of infant development* (2. Aufl., S. 192–220). Wiley-Blackwell

[CR30] Nandam LS, Brazel M, Zhou M, Jhaveri DJ (2020) Cortisol and major depressive disorder—translating findings from humans to animal models and back. Front Psychiatry 10:974. 10.3389/fpsyt.2019.00974 32038323 10.3389/fpsyt.2019.00974 PMC6987444

[CR31] Nierop A, Bratsikas A, Klinkenberg A, Nater UM, Zimmermann R, Ehlert U (2006) Prolonged salivary cortisol recovery in second-trimester pregnant women and attenuated salivary α-amylase responses to psychosocial stress in human pregnancy. J Clin Endocrinol Metab 91(4):1329–1335. 10.1210/jc.2005-1816 16434458 10.1210/jc.2005-1816

[CR32] Pearson RM, Cooper RM, Penton-Voak IS, Lightman SL, Evans J (2010) Depressive symptoms in early pregnancy disrupt attentional processing of infant emotion. Psychol Med 40(4):621–631. 10.1017/S0033291709990961 19671214 10.1017/S0033291709990961

[CR33] Pearson RM, Lightman SL, Evans J (2011) Attentional processing of infant emotion during late pregnancy and mother–infant relations after birth. Arch Womens Ment Health 14(1):23–31. 10.1007/s00737-010-0180-4 20859644 10.1007/s00737-010-0180-4

[CR34] Penner F, Rutherford HJV (2022) Emotion regulation during pregnancy: A call to action for increased research, screening, and intervention. Archives Women’s Mental Health 25(2):527–531. 10.1007/s00737-022-01204-0 10.1007/s00737-022-01204-0 PMC8748524 35015146

[CR35] Pruessner JC, Wolf OT, Hellhammer DH, Buske-Kirschbaum A, von Auer K, Jobst S, Kaspers F, Kirschbaum C (1997) Free cortisol levels after awakening: A reliable biological marker for the assessment of adrenocortical activity. Life Sci 61(26):2539–2549. 10.1016/S0024-3205(97)01008-4 9416776 10.1016/s0024-3205(97)01008-4

[CR36] Pruessner JC, Kirschbaum C, Meinlschmid G, Hellhammer DH (2003) Two formulas for computation of the area under the curve represent measures of total hormone concentration versus time-dependent change. Psychoneuroendocrinology 28(7):916–931. 10.1016/S0306-4530(02)00108-7 12892658 10.1016/s0306-4530(02)00108-7

[CR37] R Core Team (2022) *R: A Language and environment for statistical computing* [Software]. R Foundation for Statistical Computing, Vienna, Austria. https://www.R-project.org/

[CR38] Rutherford HJV, Joormann J (2017) The role of attentional bias in prenatal and postpartum depression. Women’s Health Res 1(1):1–9. 10.1057/whr0000001

[CR39] Rutherford HJV, Graber KM, Mayes LC (2016) Depression symptomatology and the neural correlates of infant face and cry perception during pregnancy. Soc Neurosci 11(4):467–474. 10.1080/17470919.2015.1108224 26465979 10.1080/17470919.2015.1108224

[CR40] Sanchez A, Vazquez C, Marker C, LeMoult J, Joormann J (2013) Attentional disengagement predicts stress recovery in depression: an eye-tracking study. J Abnorm Psychol 122(2):303–313. 10.1037/a0031529 23421524 10.1037/a0031529

[CR41] Seth S, Lewis AJ, Galbally M (2016) Perinatal maternal depression and cortisol function in pregnancy and the postpartum period: A systematic literature review. BMC Pregnancy Childbirth 16(1):124. 10.1186/s12884-016-0915-y 27245670 10.1186/s12884-016-0915-y PMC4886446

[CR42] Shin H, Park Y, Ryu H, Seomun G (2008) Maternal sensitivity: a concept analysis. J Adv Nurs 64(3):304–314. 10.1111/j.1365-2648.2008.04814.x 18764848 10.1111/j.1365-2648.2008.04814.x

[CR43] Slade A, Holland ML, Ordway MR, Carlson EA, Jeon S, Close N, Mayes LC, Sadler LS (2020) Minding the baby^®^: enhancing parental reflective functioning and infant attachment in an attachment-based, interdisciplinary home visiting program. Dev Psychopathol 32(1):123–137. 10.1017/S0954579418001463 30636649 10.1017/S0954579418001463

[CR44] Smith MV, Shao L, Howell H, Lin H, Yonkers KA (2011) Perinatal depression and birth outcomes in a healthy start project. Matern Child Health J 15(3):401–409. 10.1007/s10995-010-0595-6 20300813 10.1007/s10995-010-0595-6 PMC3757503

[CR45] Stein A, Pearson RM, Goodman SH, Rapa E, Rahman A, McCallum M, Howard LM, Pariante CM (2014) Effects of perinatal mental disorders on the fetus and child. Lancet 384(9956):1800–1819. 10.1016/S0140-6736(14)61277-0 25455250 10.1016/S0140-6736(14)61277-0

[CR46] Thompson LA, Trevathan WR (2008) Cortisol reactivity, maternal sensitivity, and learning in 3-month-old infants. Infant Behav Dev 31(1):92–106. 10.1016/j.infbeh.2007.07.007 17716739 10.1016/j.infbeh.2007.07.007 PMC2277326

[CR47] Thompson-Booth C, Viding E, Mayes LC, Rutherford HJV, Hodsoll S, McCrory E (2014a) I can’t take my eyes off of you: attentional allocation to infant, child, adolescent and adult faces in mothers and non-mothers. PLoS ONE 9(10):e109362. 10.1371/journal.pone.0109362 25353640 10.1371/journal.pone.0109362 PMC4212970

[CR48] Thompson-Booth C, Viding E, Mayes LC, Rutherford HJV, Hodsoll S, McCrory EJ (2014b) Here’s looking at you, kid: attention to infant emotional faces in mothers and non-mothers. Dev Sci 17(1):35–46. 10.1111/desc.12090 24341972 10.1111/desc.12090 PMC4352331

[CR49] Van Ens W, Schmidt U, Campbell IC, Roefs A, Werthmann J (2019) Test-retest reliability of attention bias for food: robust eye-tracking and reaction time indices. Appetite 136:86–92. 10.1016/j.appet.2019.01.020 30682381 10.1016/j.appet.2019.01.020

[CR50] De Weerth C, Gispen-de wied CC, Jansen LMC, Buitelaar JK (2007) Cardiovascular and cortisol responses to a psychological stressor during pregnancy. Acta Obstet Gynecol Scand 86(10):1181–1192. 10.1080/00016340701547442 17851798 10.1080/00016340701547442

[CR51] Xu Y, Zheng P, Feng W, Chen L, Sun S, Liu J, Tang W, Bao C, Xu L, Xu D, Zhao K (2023) Patterns of attentional bias in antenatal depression: an eye-tracking study. Front Behav Neurosci 17:1288616. 10.3389/fnbeh.2023.1288616 38192488 10.3389/fnbeh.2023.1288616 PMC10773570

[CR52] Yin X, Sun N, Jiang N, Xu X, Gan Y, Zhang J, Qiu L, Yang C, Shi X, Chang J, Gong Y (2021) Prevalence and associated factors of antenatal depression: systematic reviews and meta-analyses. Clin Psychol Rev 83:101932. 10.1016/j.cpr.2020.101932 33176244 10.1016/j.cpr.2020.101932

[CR53] Zhang X, Han ZR, Gatzke-Kopp LM (2021) A biopsychosocial approach to emotion-related parenting: physiological responses to child frustration among urban Chinese parents. J Fam Psychol 35(5):639–648. 10.1037/fam0000824 33705175 10.1037/fam0000824

[CR54] Zorn JV, Schür RR, Boks MP, Kahn RS, Joëls M, Vinkers CH (2017) Cortisol stress reactivity across psychiatric disorders: a systematic review and meta-analysis. Psychoneuroendocrinology 77:25–36. 10.1016/j.psyneuen.2016.11.036 28012291 10.1016/j.psyneuen.2016.11.036

