# Supplementary material for: Associations between attentional disengagement from distressed infant faces and cortisol reactivity are moderated by depressive symptoms in pregnant women: an eye-tracking study
Source: Arch Womens Ment Health. 2026 Jan 3;29(1):3. doi: 10.1007/s00737-025-01638-2 (PMC12764579; doi:10.1007/s00737-025-01638-2)
Supplement: Supplementary file 1 — Supplementary material 1(DOCX 298 KB) [file 737_2025_1638_MOESM1_ESM.docx]

**Supplementary Material to Manuscript:**

**Associations between attentional disengagement from distressed infant faces and cortisol reactivity are moderated by depressive symptoms in pregnant women:**

**An eye-tracking study**

**Appendix A: Excluded participants**

**Details on excluded participants**

A sample of 92 participants completed both the eye-tracking and stress task. Data from three (3.26%) participants had to be removed due to a violation of study criteria (violation of age or language criterion) and 2 (2.17%) individuals had to be excluded due to issues in the procedure of the stress manipulation task (e.g., arithmetic task had to be stopped earlier). Further, for the adult eye-tracking task (focused on adult stimuli), n=13 (14.13%) participants had to be excluded (tracking ratio ≤ 65%: n=9; issues with eye-tracking task (e.g., participant had uncorrected vision): n=4). For the infant eye-tracking task (focused on infant stimuli), n=13 (14.13%) individuals were removed from the data (tracking ratio ≤ 65%: n=8; issues with eye-tracking task: n=5). Eight of these exclusions were overlapping between both tasks resulting in a final analytical sample of N=79 participants including n=36 pregnant and n=43 nulliparous women.

We tested for differences in demographic characteristics and study variables between included (n=79) and fully excluded participants (n=13). T-tests revealed no significant differences between groups, except for race (race: χ^2^(4)=18.14, *p*=.001; age: *t*(9)=-0.58, *p*=.577; ethnicity: χ^2^(1)=0.46, *p*=.500; education: χ^2^(3)=2.14, *p*=.544; marital status: χ^2^(2)=1.30, *p*=.523; pregnancy week (only pregnant participants included): *t*(2)=0.34, *p*=.764; primiparous (only pregnant participants included): χ^2^(1) < .001, *p*=1.00; AUCi cortisol: *t*(5)=-0.95, *p*=.387; Disengagement sad adult faces: *t*(14)=-0.86, *p*=0.406; Disengagement angry adult faces: *t*(30)=0.57, *p*=0.576; Disengagement happy adult faces: *t*(12)=-1.10, *p*=0.292; Disengagement distressed infant faces: *t*(19)=-0.55, *p*=.590; Disengagement happy infant faces: *t*(12)=-0.84, *p*=.416; BDI: *t*(4)=0.319, *p*=.768). However, posthoc pairwise comparisons (Bonferroni correction) performed on the variable race did not result in any significant pairwise differences (all *p* > .76).

**Appendix B: Study procedure**

**Figure S1**

*
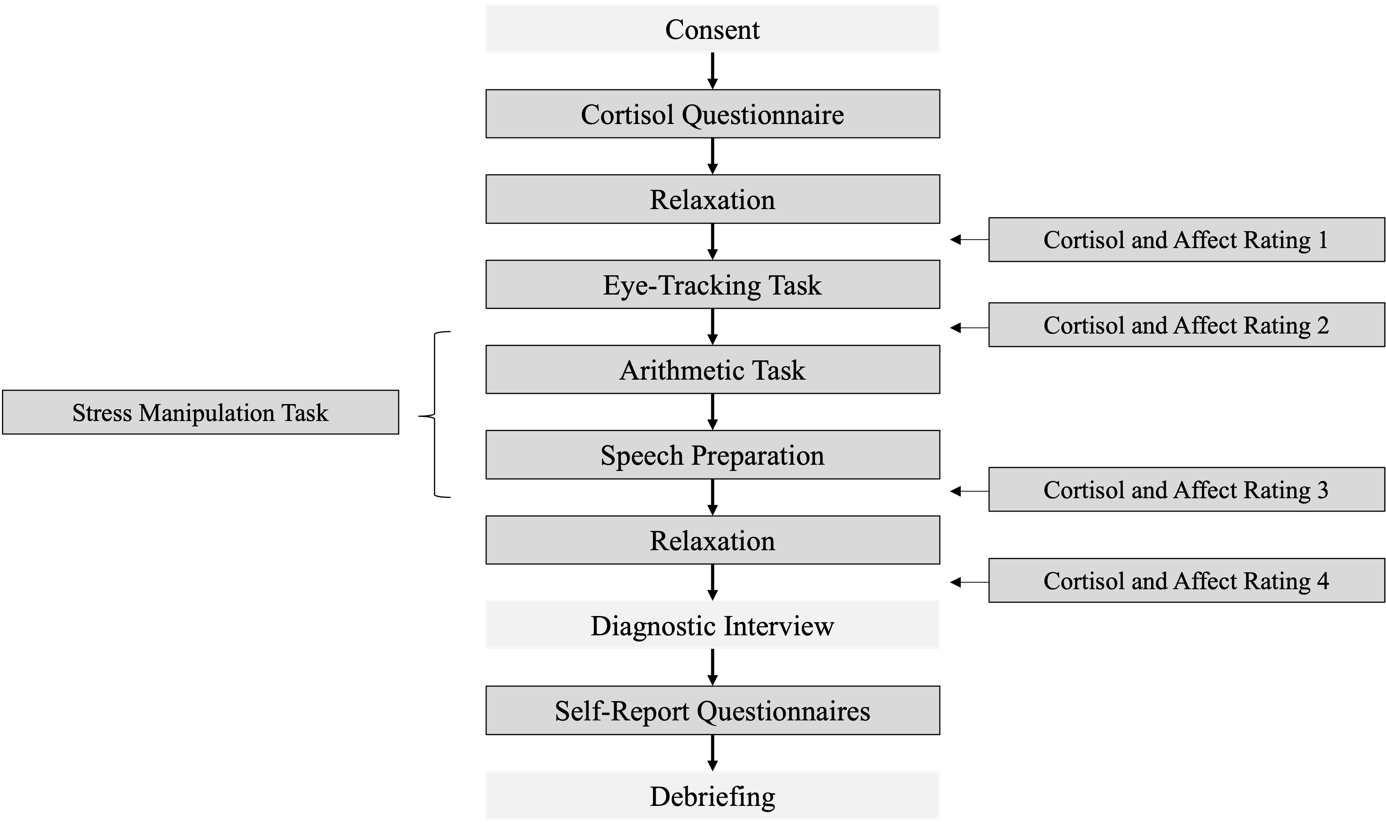
Study procedure*

*Note.* Highlighted assessments were part of the current investigation.

**Appendix C: Eye-Tracking Task**

**Detailed description of the eye-tracking task**

To assess attentional disengagement, participants completed an eye-tracking task developed by Sanchez and colleagues (2013), that in its original form focused on adult stimuli only. We used two variations of this task: an adult and an infant version. The order of which version of the task was presented to participants first was counterbalanced across individuals. For each version of the task, participants completed three different types of trials: natural viewing trials, attentional engagement trials, and attentional disengagement trials, which were presented in random order. Given we were only interested in attentional disengagement, the following descriptions focus on this type of trial only. Here, we will describe only the most important aspects of the task; a detailed description of the task can be found in the supplementary material.

**Adult version.**

***Stimuli.*** Each stimulus was composed of two pictures with one showing a face with a neutral expression and another showing a face with an emotional expression of the same individual. Face images were selected from the Karolinska Directed Emotional Faces (KDEF) database (Lundqvist et al., 1998). A total of 108 pairs of face images were used as stimuli consisting of 36 sad, 36 happy and 36 angry faces (18 male and 18 female faces for each emotion condition). Stimuli were edited to omit the neck, hair, and background of the original images in order to present only study-relevant aspects of the images. The size of adult facial stimuli was approximately 24 cm x 17.5 cm and the visual angle of each face was 22.62° (H) x 16.59° (W). The facial stimuli were 16.13° apart.

***Disengagement trials.*** At the beginning of the task, participants were asked to complete six practice trials followed by 36 trials of stimulus presentation. Figure 2 depicts the sequence of the task. Within each trial, participants were shown a white screen for 500 ms, followed by a fixation cross for an additional 500 ms. After the presentation of the fixation cross, a random number was presented at the center of the screen for 1,000 ms and participants were instructed to say the number aloud as quickly as possible in order to ensure their eye gaze was in the middle of the screen prior to stimulus presentation. After that, a pair of two faces (happy-neutral, sad-neutral, angry-neutral) were presented for 3,000 ms side by side on the screen and the eye tracker waited for a fixation of at least 100 ms on the emotionally-valenced face. The circle or square then surrounded the neutral face and participants were instructed to respond with a key press whether the face was surrounded by a square or circle as quickly as possible. The required response was used in these trials to effectively disengage participants’ attention away from the emotionally-valenced face to the neutral face. Across trials, both types of frames, square and circle, were equally likely to appear in the left or right side of the screen in all conditions.

***Eye-tracking apparatus.*** Participants’ eye movements were recorded using a Tobii TX-120 eye tracking system. The system sampled participants’ eye movements at a rate of 60 Hz. The eye tracking system was automatically synchronized to E-Prime 2.1 software at the beginning of each trial. E-prime 2.1 also controlled both stimuli presentation and coordinated the ‘wait for fixation’ period with the eye tracking system, which allowed for the task to not continue until a certain time of participant fixation had passed (100 ms). Participants were kept at a distance of 60 cm from the computer screen throughout all trials. The stimuli were presented to participants on a 100 x 150 cm screen. Participants’ eye gaze was calibrated before the start of the task.

***Attention Indices.*** Eye movements recorded by the Tobii TX-120 eye-tracking system were converted to visual fixation data using Tobii Studio software. Visual fixations were defined at a minimum of 100 ms in duration and at a maximum of 1-degree fixation radius. In this study, we were interested in attentional disengagement defined as the time it took for participants to shift their gaze from the emotional face to the neutral face after it was surrounded by either a square or circle (stage 6 in Figure 2). Included in these times was the amount of time that participants spent attending to the stimuli during the ‘wait for fixation’ period, in addition to the time it took participants to shift their gaze from the initially fixated face to the face surrounded by the square or circle.

In order to qualify as a shift in gaze to the face surrounded by square or circle on each trial, the following criteria had to be met: 1) participants must have been fixated on the opposite face before the circle or square appeared, 2) the shift in gaze eye movement must have occurred at least 100 ms after the circle or square appeared, 3) the shift in gaze must have been directed to the face surrounded by the square or circle, and 4) the participant must have fixated on the stimuli for at least 100 ms after shifting their gaze to it.

For our analyses, we used average scores for attentional disengagement for each emotion condition for each participant (e.g., the average time that it took for a participant to shift their gaze from sad adult faces to neutral adult faces across all valid trials, in seconds).

**Infant version.** To assess attentional disengagement from infant faces, we used the same procedure as for the adult task developed by Sanchez and colleagues (Sanchez et al., 2013) but adapted it by using infant facial stimuli and only two different emotional conditions (happy and distressed faces). The task, the eye-tracking apparatus as well as the attention indices used were the same as for the adult task.

***Stimuli.*** Infant face stimuli were extracted from a database consisting of digital photographs of 27 infants (Kringelbach et al., 2008). 66 pairs of face images were used consisting of 33 happy and 33 distressed faces. Stimuli were edited to omit the neck, hair, and background of the original images in order to present only study-relevant aspects of the images. The size of the infant facial stimuli was approximately 23 cm x 19 cm with a visual angle of 21.7° (H) x 17.99° (W). The faces were 15.19° apart.

**Figure S2**

*
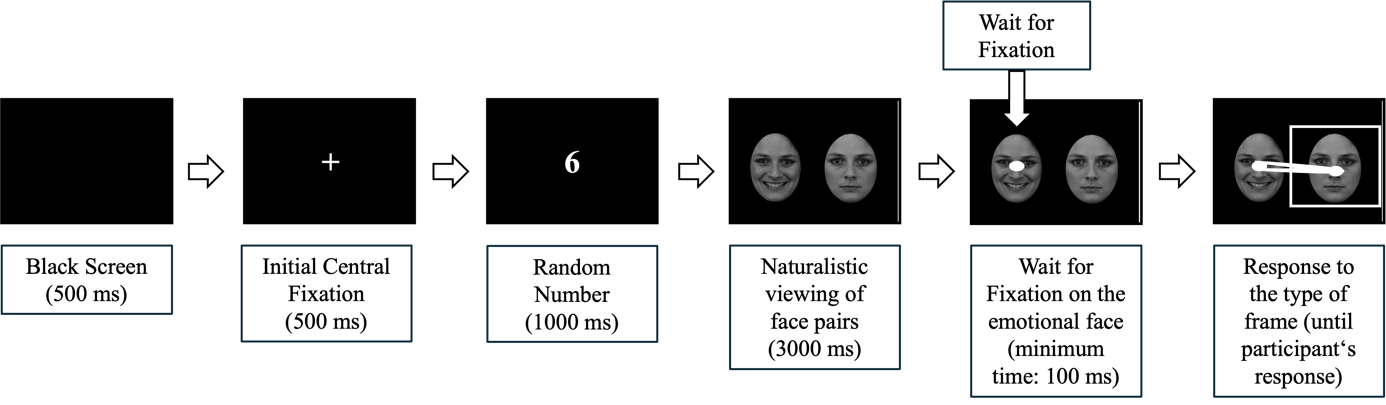
Representation of the sequence of disengagement trials*

*Note.* Figure adapted from Sanchez et al. (2013).

**Appendix D: Cortisol and Saliva Samples**

**Additional information on storing and analysis of saliva samples**

Saliva samples were stored in a freezer at -80 degree Celsius and analyzed at the Immune Monitoring Core Facility at the Yale School of Medicine using a multiplex assay method (Milliplex high-sensitivity assays; Millipore Sigma).

**Figure S3**

*Timing of cortisol assessments*

*
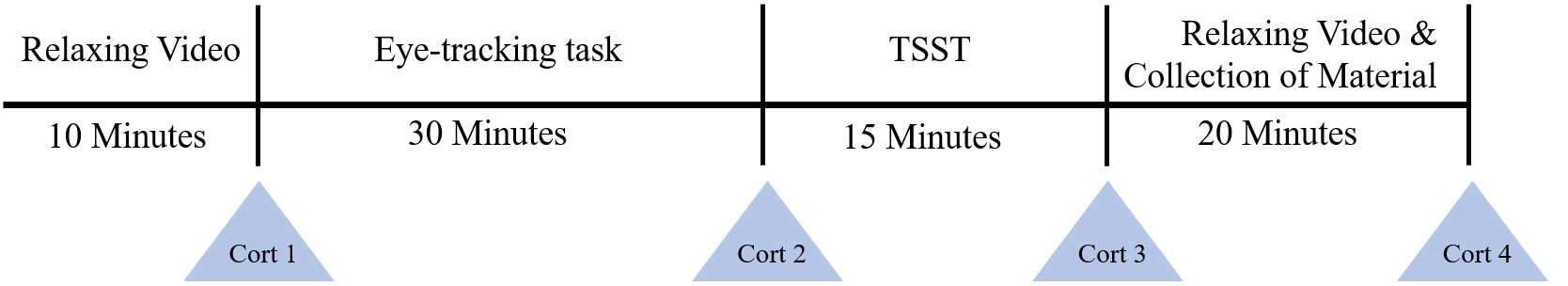
*

*Note.* Cort=Cortisol.

**Additional information on calculation of AUCi scores**

Calculation of AUCi scores was based on the following formula:

$$AUCg =\frac{time interval 1(Cort1+Cort2)}{2}+\frac{time interval 2(Cort2+Cort3)}{2}+\frac{time interval 3(Cort3+Cort4)}{2}$$

$$AUCi =AUCg-Cort1(time interval 1 +time interval 2 +time interval 3)$$

**Cortisol confounders**

Based on previous recommendations regarding confounders in cortisol assessments (Narvaez Linares et al., 2020), participants were asked to fill out a questionnaire on potential cortisol confounders on the day of the session (use of hormonal birth control, use of other medication, mouth injuries, caffeine and tobacco use). T-tests revealed no significant differences in AUCi cortisol levels between those indicating potential confounders and those who did not (*t*-tests were conducted for those confounders with a sufficient number of affirmative responses): hormonal birth control: *t*(16)=1.45, *p*=.17; other medication: *t*(52)=1.21, *p*=0.23; caffeine: *t*(71)=-0.88, *p*=0.38; tobacco: *t*(2)=-1.19, *p*=0.34.

**Appendix E: Psychiatric status of participants**

**Additional questionnaires**

To better assess the psychiatric status of participants, we also administered the Beck Anxiety Inventory (BAI; Beck & Steer, 1990) as well as the depression modules of the Structured Clinical Interview for DSM-IV axis I disorders (SCID-I; First et al., 1997). Descriptives on these measures can be found below.

**Table S1**

*Distribution of the BAI*

| M (SD) | Range | n (%) of participants meeting clinical classifications | | | | |
| --- | --- | --- | --- | --- | --- | --- |
|  |  | Minimal anxiety | Mild anxiety | Moderate anxiety | Severe anxiety | Clinical cut-off anxiety (score ≥16) |
| 7.28 (6.38) | 0-32 | 39(65%) | 14 (23%) | 6 (10%) | 1 (2%) | 7 (12%) |

*Note.* Clinical classifications based on Beck & Steer (1993). BAI data were available for n=60 participants.

**Table S2**

*Number of participants fulfilling criteria for depression diagnoses based on SCID-I depression modules*

|  | PDD past | PDD current | MDD past | MDD past recurring | MDD current |
| --- | --- | --- | --- | --- | --- |
| n (%) | 0 (0%) | 2 (5%) | 13 (33%) | 0 (0%) | 1 (3%) |

*Note.* Clinical classification based on First et al. (1997). Data were available for n=39 participants. PDD=Persistent Depressive Disorder; MDD=Major Depressive Disorder.

**Appendix F: Sensitivity analyses controlling for time of day**

**Table S3**

*Results of linear regression models investigating associations between attentional disengagement from adult faces, depression, and cortisol reactivity in the full sample (N=79) controlling for time of day of lab sessions*

|  | Predicted | Cortisol Reactivity | | | |
| --- | --- | --- | --- | --- | --- |
|  |  | *B* | *SE* | *t* | 95% *CI* |
| Sad faces | Intercept | -60.92* | 26.41 | -2.31 | -113.80, -8.05 |
|  | Disengagement | 180.69 | 155.61 | 1.16 | -130.91, 492.29 |
|  | Depressive symptoms | 0.70 | 3.03 | 0.23 | -5.36, 6.77 |
|  | Disengagement:Depressive symptoms | -18.76 | 31.10 | -0.60 | -81.05, 43.52 |
| Angry faces | Intercept | -65.02* | 27.89 | -2.33 | -120.83, -9.21 |
|  | Disengagement | 33.57 | 127.04 | 0.26 | -220.64, 287.78 |
|  | Depressive symptoms | 1.29 | 3.16 | 0.41 | -5.04, 7.61 |
|  | Disengagement:Depressive symptoms | 5.99 | 18.34 | 0.33 | -30.72, 42.69 |
| Happy faces | Intercept | -66.49* | 27.65 | -2.41 | -121.83, -11.15 |
|  | Disengagement | -37.09 | 223.66 | -0.17 | -484.79, 410.60 |
|  | Depressive symptoms | 0.68 | 3.04 | 0.23 | -5.40, 6.77 |
|  | Disengagement:Depressive symptoms | -0.96 | 48.48 | -0.02 | -97.99, 96.07 |

*Note.* Time of day variable included as a categorical variable with three categories, namely (1) before 11:30, (2) between 11:30 and 2:00, and (3) after 2:00; **p* < .05

**Table S4**

*Results of linear regression models investigating associations between attentional disengagement from infant faces, depression, and cortisol reactivity in the pregnant group (n=36) controlling for time of day of lab sessions*

|  | Predicted | Cortisol Reactivity | | | | Cortisol Reactivity | | | |
| --- | --- | --- | --- | --- | --- | --- | --- | --- | --- |
|  |  | Model: Step 1 | | | | Model: Step 2 | | | |
|  |  | *B* | *SE* | *t* | 95% *CI* | *B* | *SE* | *t* | 95% *CI* |
| Distressed faces | Intercept | 15.63 | 34.82 | 0.45 | -55.39, 86.65 | 48.38 | 30.07 | 1.61 | -13.68, 110.45 |
|  | Disengagement | -206.80* | 92.73 | -2.23 | 395.91, -17.68 | 115.73 | 141.49 | 0.82 | -176.29, 407.75 |
|  | Depressive symptoms |  |  |  |  | 0.06 | 4.92 | 0.01 | -10.09, 10.22 |
|  | Disengagement:Depressive symptoms |  |  |  |  | 123.47** | 43.76 | 2.82 | 33.15, 213.79 |
| Happy faces | Intercept | -10.05 | 35.46 | -0.28 | -82.46, 62.35 | -12.35 | 34.37 | -0.36 | -83.44, 58.75 |
|  | Disengagement | 154.01 | 92.67 | 1.66 | -35.26, 343.27 | 98.69 | 124.09 | 0.80 | -158.01, 355.38 |
|  | Depressive symptoms |  |  |  |  | 2.26 | 6.11 | 0.37 | -10.39, 14.90 |
|  | Disengagement:Depressive symptoms |  |  |  |  | -23.63 | 41.54 | -0.57 | -109.56, 62.30 |

*Note.* In Step 1 of the model, disengagement was entered as the only predictor. In Step 2, depressive symptoms and interaction of depressive symptoms and disengagement were added. Time of day variable included as a categorical variable with three categories, namely (1) before 11:30, (2) between 11:30 and 2:00, and (3) after 2:00; **p* < .05; ***p* < .01.

**Table S5**

*Results of linear regression models investigating associations between attentional disengagement from infant faces, depression, and cortisol reactivity in the nulliparous group (n=43) controlling for time of day of lab sessions*

|  | Predicted | Cortisol Reactivity | | | | Cortisol Reactivity | | | |
| --- | --- | --- | --- | --- | --- | --- | --- | --- | --- |
|  |  | Model: Step 1 | | | | Model: Step 2 | | | |
|  |  | *B* | *SE* | *t* | 95% *CI* | *B* | *SE* | *t* | 95% *CI* |
| Distressed faces | Intercept | -88.64** | 27.61 | -3.21 | -144.76, -32.53 | -93.96** | 31.82 | -2.95 | -159.25, -28.67 |
|  | Disengagement | 23.18 | 70.16 | 0.33 | -119.40, 165.77 | 31.61 | 90.67 | 0.35 | -154.43, 217.65 |
|  | Depressive symptoms |  |  |  |  | -0.47 | 3.50 | -0.14 | -7.66, 6.71 |
|  | Disengagement:Depressive symptoms |  |  |  |  | 3.54 | 29.48 | 0.12 | -56.95, 64.02 |
| Happy faces | Intercept | -92.77** | 28.93 | -3.21 | -151.69, -33.85 | -100.59** | 32.73 | -3.07 | -167.99, -33.19 |
|  | Disengagement | 71.48 | 145.78 | 0.49 | -225.46, 368.42 | 114.03 | 165.68 | 0.69 | -227.20, 455.25 |
|  | Depressive symptoms |  |  |  |  | 0.10 | 3.24 | 0.03 | -6.58, 6.77 |
|  | Disengagement:Depressive symptoms |  |  |  |  | 30.48 | 28.71 | 1.06 | -28.64, 89.60 |

*Note.* In Step 1 of the model, disengagement was entered as the only predictor. In Step 2, depressive symptoms and interaction of depressive symptoms and disengagement were added. Time of day variable included as a categorical variable with three categories, namely (1) before 11:30, (2) between 11:30 and 2:00, and (3) after 2:00; ***p* < .01.

**Appendix G: Correlations among study variables**

**Table S6**

*Correlations among study variables across the full sample (N=79)*

|  |  | Cortisol reactivity | Depressive symptoms | Disengagement from adult faces | | | | Disengagement from infant faces | | |
| --- | --- | --- | --- | --- | --- | --- | --- | --- | --- | --- |
|  |  |  |  | Sad | Angry | | Happy | | Distressed | Happy |
| Cortisol reactivity |  | 1 | .06 | .12 | .05 | -.00 | | | -.12 | .18 |
| Depressive symptoms |  |  | 1 | .08 | -.16 | .00 | | | -.10 | -.08 |
| Disengagement from adult faces | Sad |  |  | 1 | -.00 | .31** | | | .15 | .41*** |
|  | Angry |  |  |  | 1 | .07 | | | .09 | .17 |
|  | Happy |  |  |  |  | 1 | | | .12 | .06 |
| Disengagement from infant faces | Distressed |  |  |  |  |  | | | 1 | -.07 |
|  | Happy |  |  |  |  |  | | |  | 1 |

*Note.* This table shows correlations among all study variables using the Pearson correlation coefficient; ***p* < .01; ****p* < .001

**References**

Beck, A. T., & Steer, R. A. (1990). *Manual for the Beck Anxiety Inventory*. Psychological Corporation.

First, M. B., Spitzer, R. L., Gibbon, M., & Williams, J. B. W. (1997). *Structured Clinical Interview for DSM-IV Axis I Disorders (SCID I).* Biometric Research Department.

Kringelbach, M. L., Lehtonen, A., Squire, S., Harvey, A. G., Craske, M. G., Holliday, I. E., Green, A. L., Aziz, T. Z., Hansen, P. C., Cornelissen, P. L., & Stein, A. (2008). A Specific and Rapid Neural Signature for Parental Instinct. *PLoS ONE*, *3*(2), e1664. https://doi.org/10.1371/journal.pone.0001664

Linares, N. N., Charron, V., Ouimet, A. J., Labelle, P. R., & Plamondon, H. (2020). A systematic review of the Trier Social Stress Test methodology: Issues in promoting study comparison and replicable research. *Neurobiology of stress*, *13*, 100235. https://doi.org/10.1016/j.ynstr.2020.100235

Lundqvist, D., Flykt, A., & Öhman, A. (1998). *The Karolinska Directed Emotional Faces (KDEF).* Department of Neurosciences Karolinska Hospital.

Sanchez, A., Vazquez, C., Marker, C., LeMoult, J., & Joormann, J. (2013). Attentional disengagement predicts stress recovery in depression: An eye-tracking study. *Journal of Abnormal Psychology*, *122*(2), 303–313. https://doi.org/10.1037/a0031529
